# Treatment patterns and survival in HER2-positive early breast cancer: a whole-of-population Australian cohort study (2007–2016)

**DOI:** 10.1038/s41416-019-0612-5

**Published:** 2019-11-01

**Authors:** Monica Tang, Andrea Schaffer, Belinda E. Kiely, Benjamin Daniels, Robert J. Simes, Chee K. Lee, Sallie-Anne Pearson

**Affiliations:** 1 Centre for Big Data Research in Health, University of New South Wales, Level 4, Lowy Building (C25), Corner Botany and High Streets, UNSW Sydney, NSW 2052 Australia; 2 NHMRC Clinical Trials Centre, University of Sydney, Levels 4-6 Medical Foundation Building, 92-94 Parramatta Rd, Camperdown, NSW 2050 Australia

**Keywords:** Breast cancer, Targeted therapies, Translational research

## Abstract

**Background:**

Randomised clinical trials (RCTs) demonstrate that trastuzumab improves survival in patients with human epidermal growth factor 2-positive early breast cancer (HER2 + EBC), but real-world patients and clinical practice often differ from RCTs. We examine real-world treatment patterns and outcomes associated with trastuzumab for HER2 + EBC.

**Methods:**

We identified all Australians dispensed trastuzumab for HER2 + EBC between 1/1/2007 and 30/6/2016. We estimated the proportion of patients completing 12 months of treatment (defined as ≥350 days of exposure within 540 days of initiation). We estimated overall survival (OS) and recurrence-free survival (RFS) by using trastuzumab dispensing for metastatic breast cancer as a surrogate for recurrence.

**Results:**

Our study included 14,644 patients. Among patients with ≥540 days of follow-up (*n* = 11,903), 67.4% completed 12 months of trastuzumab. OS rates at 5 and 9 years were 92.7 and 87.9%, and RFS rates at 5 and 9 years were 86.8 and 81.4%, respectively. Patients who completed 12 months of trastuzumab had a 9-year OS rate of 90.2% compared with 86.2% among patients receiving <12 months of therapy (adjusted HR 0.71, 95% CI 0.62–0.81).

**Conclusions:**

Real-world HER2 + EBC patients are less likely to complete 12 months of trastuzumab than some clinical trial counterparts but have survival outcomes comparable to those reported in landmark RCTs.

## Background

Human epidermal growth factor 2 (HER2)-positive breast cancers are characterised by amplification of the HER2 gene or overexpression of the HER2 protein and account for 15–20% of breast malignancies.^1^ The introduction of HER2-targeted therapies has changed the treatment paradigm of HER2-positive cancers, which were previously associated with more aggressive disease and poorer outcomes.^2,3^

Trastuzumab was the first targeted therapy developed to treat HER2-positive breast cancers. Several randomised controlled trials (RCTs) demonstrated that trastuzumab in addition to adjuvant chemotherapy improves disease-free survival (DFS) and overall survival (OS) in women with HER2-positive early breast cancer.^4–11^ The majority of patients in the landmark RCTs received 12 months of adjuvant trastuzumab.^4,6,10^ As subsequent studies investigating the efficacy of shorter durations of adjuvant trastuzumab are conflicting,^12–20^ 12 months of trastuzumab remains the standard of care for HER2-positive early breast cancer. As the evidence for prescribing adjuvant trastuzumab is the strongest for high-risk cancers,^4,6,10,11^ not all early breast cancers underwent HER2 testing when trastuzumab was first introduced,^21^ and not all HER2-positive early breast cancers are treated with trastuzumab.^21–23^

The strict eligibility criteria of RCTs limit their generalisability to patients treated in the clinic. Trial participants tend to have fewer adverse events and lower mortality rates than non-participants due to differences in their baseline characteristics and comorbidities.^24–28^ Therefore, data generated from patients treated in routine care are instrumental in assessing whether RCT outcomes translate to the real-world setting. According to previous real-world studies, up to one-third of patients treated with adjuvant trastuzumab do not receive a standard, guideline-recommended chemotherapy regimen,^29,30^ and up to 23% do not complete the recommended 12-month course of trastuzumab.^31–35^

By virtue of Australia’s universal health system, data are available on all patients who accessed trastuzumab for breast cancer since its public subsidy in 2006. In this whole-of-population study, we describe treatment patterns and outcomes associated with adjuvant trastuzumab for early breast cancer. Specifically, we describe patient characteristics, trastuzumab completion rates, survival and cancer recurrence outcomes, and the association of trastuzumab completion with outcomes.

## Methods

### Study setting

In Australia, all citizens and permanent residents have government-subsidised access to prescription medicines listed on the Pharmaceutical Benefits Scheme (PBS). Trastuzumab has been PBS-listed for the adjuvant treatment of HER2-positive early breast cancer, irrespective of size and nodal status, since 1 October 2006. Trastuzumab for metastatic breast cancer was funded through the Herceptin Program, outside the PBS, from December 2001 until July 2015, when the Herceptin Program was discontinued and trastuzumab was PBS-listed for all breast cancer stages. We have previously described this whole-of-population cohort of HER2-positive breast cancer patients.^36^ Private insurance in Australia does not reimburse publicly subsidised medicines, so our study population likely captures all Australian residents receiving trastuzumab for HER2-positive early breast cancer.

### Data sources

We conducted a retrospective, population-based, cohort study of all Australians with HER2-positve early breast cancer who received PBS trastuzumab in the adjuvant and neoadjuvant settings. The patient dataset includes sex, year of birth, month/year of death, remoteness of residence and socioeconomic disadvantage. Fact of death data was supplied in month/year format through linkage to the National Death Index. Cause of death was not available, so it was not possible to estimate breast cancer-specific survival. PBS-dispensing records comprise dispensing date, quantity dispensed and PBS-item number, which identifies the indication for which a medicine is prescribed (e.g. early or metastatic HER2-positive breast cancer) and the specific regimen (e.g. weekly or 3-weekly dosing) (Supplementary Table 1).

### Study population

We included all patients who received ≥1 dispensing of PBS-subsidised trastuzumab for HER2-positive early breast cancer between 1 January 2007 and 30 June 2016. We excluded patients who initiated trastuzumab within the first 3 months of PBS listing of trastuzumab for early breast cancer (1 October–31 December 2006, *n* = 897) as they may have commenced trastuzumab outside the PBS before its public subsidy. We also excluded patients who were dispensed trastuzumab for metastatic HER2-positive breast cancer prior to receiving trastuzumab for early HER2-positive breast cancer (*n* = 320).

### Trastuzumab use

We identified trastuzumab use based on Anatomical Therapeutic Chemical Classification System (ATC) code L01XC03 and PBS-item numbers for early breast cancer.

We assigned 7 or 21 days of exposure to each trastuzumab dispensing based on PBS-item codes for weekly or 3-weekly dosing regimens. We summed the number of days of trastuzumab exposure over all dispensings for each patient. To allow for treatment breaks, we defined completion of a 12-month course of adjuvant trastuzumab as having ≥350 days of trastuzumab exposure within 540 days of trastuzumab initiation. We also examined patients with ≥270, ≥165 or ≥80 days of trastuzumab exposure. We selected 540 days as the observation time to assess trastuzumab completion to account for patients who may take longer than 12 months to complete a full course of adjuvant trastuzumab due to treatment holidays or concurrent illness; this definition has been used in previous studies of adjuvant trastuzumab using health administrative data.^34,35^ To determine completion rates, we restricted the population to patients with ≥540 days of follow-up (i.e. initiated trastuzumab between 1 January 2007 and 31 December 2014) as this was the observation time used to assess completion of an entire course of adjuvant trastuzumab. We used logistic regression to identify baseline characteristics associated with completion of trastuzumab. We performed sensitivity analyses whereby we defined completion as having ≥350 days of trastuzumab exposure within 365 or 450 days of trastuzumab initiation.

### Concomitant chemotherapy and endocrine therapy

We determined commonly prescribed (neo)adjuvant chemotherapy regimens based on the dispensing of cytotoxic agents, identified by ATC codes commencing with L01, in the period 120 days before until 60 days after the first trastuzumab dispensing. We defined oestrogen receptor (ER)-positive patients as those who were dispensed endocrine therapy within 120 days before or 540 days after the first trastuzumab dispensing.^37^ We identified patients who received a selective oestrogen receptor modulator (tamoxifen or toremifene) and/or aromatase inhibitor (anastrozole, letrozole or exemestane) by using ATC codes.

### Outcomes

We calculated OS as the time between the first dispensing of adjuvant trastuzumab to the date of death, set as the last day of each month, as fact of death data was supplied in month and year format. All individuals had follow-up for date of death data until 31 May 2017. We used Kaplan–Meier curves and Cox regression analysis to examine factors associated with OS, including baseline characteristics and completion of trastuzumab course. When examining associations between trastuzumab completion and OS, we excluded patients who died within 540 days of trastuzumab initiation and did not have the opportunity to receive a full 12-month course.^38^

We used the date of the first trastuzumab dispensing for metastatic disease as a surrogate for recurrence of HER2-positive breast cancer, based on PBS-dispensing records available until 30 June 2016. We defined time to recurrence as the time between the first dispensing of adjuvant trastuzumab to the first trastuzumab dispensing for metastatic disease. We calculated recurrence-free survival (RFS) from the date of the first adjuvant trastuzumab dispensing to the date of recurrence or death.

For patients with at least 5 years of dispensing data follow-up (i.e. the first trastuzumab dispensing between 1 January 2007 and 30 June 2011), we calculated the number of recurrences occurring in each year of follow-up.

### Ethics and data access

This study was approved by the NSW Population and Health Services Research Ethics Committee (Approval Number: 2010/02/213). The Australian Department of Human Services (DHS) External Request Evaluation Committee approved access to the data (Approval Numbers: MI1474, MI1475, MI1477 and MI5858).

## Results

### Study population

Between 1 October 2006 and 30 June 2016, 15,861 Australians were dispensed at least one dose of (neo)adjuvant trastuzumab for HER2-positive early breast cancer. Of these, 14,644 patients received their first trastuzumab dispensing between 1 January 2007 and 30 June 2016 and were included in our study (Table 1). Median age at the first trastuzumab dispensing was 55 years (range 22–94, interquartile range [IQR] 47–74). Most of them resided in a capital city (62.7%), and patients from the quintile with the most socioeconomic disadvantage were underrepresented (12.7%).

**Table 1:** Baseline characteristics of study population of all patients dispensed trastuzumab from 2007 to 2016 (*n* = 14,644)

	Frequency	(%)
Age at the first trastuzumab dispensing		
≤30 years	179	1.2
31–40 years	1354	9.3
41–50 years	3661	25
51–60 years	4558	31.1
61–70 years	3333	22.8
71–80 years	1349	9.2
>80 years	210	1.4
Sex		
Female	14,590	99.6
Male	54	0.4
Year of the first trastuzumab dispensing		
2007–2008	2353	16.1
2009–2010	2823	19.3
2011–2012	3068	21
2013–2014	3659	25
2015–2016	2741	18.7
Remoteness		
Capital cities	9177	62.7
Other metropolitan centres	1155	7.9
Large rural centres	965	6.6
Other rural	2984	20.4
Remote	238	1.6
Missing	125	0.9
State		
New South Wales	4892	33.4
Australian Capital Territory	210	1.4
Northern Territory	122	0.8
Queensland	2890	19.7
South Australia	1098	7.5
Tasmania	304	2.1
Victoria	3864	26.4
Western Australia	1264	8.6
Index of Relative Socioeconomic Disadvantage		
1 (most disadvantage)	1862	12.7
2	3201	21.9
3	2873	19.6
4	3413	23.3
5 (least disadvantage)	3234	22.1
Missing	61	0.4

### Trastuzumab use

Patients initiating adjuvant trastuzumab between 1 January 2007 and 30 June 2016 (*n* = 14,644) had a median of 17 trastuzumab dispensings (IQR 15–18).

Among patients with at least 540 days of follow-up (*n* = 11,903), 8018 (67.4%) completed 12 months of adjuvant trastuzumab (i.e. were dispensed ≥350 days of trastuzumab). 21.9% received 270–349 days of therapy, 5.6% received 165–269 days, 3.3% received 80–164 days and 1.8% received <80 days. Age >80, socioeconomic disadvantage and residing outside metropolitan areas were associated with lower likelihood of completing trastuzumab (Table 2). Patients initiating trastuzumab in later years were more likely to complete a 12-month course compared with those initiating in 2007–2008. In our sensitivity analyses, 58.0% of patients completed 12 months of trastuzumab within 365 days of initiation, and 66.3% of patients completed 12 months of trastuzumab within 450 days of initiation.

**Table 2:** Baseline factors associated with completion of treatment (logistic regression) in patients with at least 540 days of follow-up from the first trastuzumab dispensing (i.e. initiated trastuzumab 2007–2014, *n* = 11903)

	Completed the course	Did not complete the course	Univariate analysis		Multivariable analysis	
			Odds ratio	95% CI	Odds ratio	95% CI
Age category						
≤30 years	102	46	1	Reference	1	
31–40 years	757	355	0.96	0.66–1.39	0.96	0.66–1.40
41–50 years	2068	942	0.99	0.69–1.41	1.02	0.71–1.46
51–60 years	2552	1206	0.95	0.67–1.36	0.99	0.69–1.42
61–70 years	1819	891	0.92	0.64–1.32	0.95	0.66–1.37
71–80 years	646	372	0.78	0.54–1.14	0.77	0.53–1.12
80+ years	74	73	0.46	0.28–0.74	0.42	0.26–0.68
State						
New South Wales	2764	1185	1		1	
Australian Capital Territory	98	66	0.64	0.46–0.87	0.53	0.38–0.74
Northern Territory	64	41	0.67	0.45–1.00	0.82	0.54–1.25
Queensland	1531	826	0.8	0.71–0.89	0.85	0.76–0.96
South Australia	504	396	0.55	0.47–0.63	0.54	0.47–0.63
Tasmania	152	89	0.73	0.56–0.96	0.75	0.57–0.99
Victoria	2231	936	1.02	0.92–1.13	1.02	0.91–1.13
Western Australia	674	346	0.84	0.72–0.97	0.81	0.69–0.94
Index of relative socioeconomic disadvantage						
1 (most disadvantage)	946	529	1		1	
2	1711	905	1.06	0.93–1.21	1.05	0.92–1.21
3	1549	797	1.09	0.95–1.25	1.03	0.89–1.19
4	1921	879	1.22	1.07–1.40	1.13	0.98–1.29
5 (least disadvantage)	1869	761	1.37	1.20–1.57	1.23	1.06–1.42
Missing	22	14	0.88	0.45–1.73	0.74	0.36–1.51
Remoteness						
Capital cities	5204	2283	1		1	
Other metropolitan centres	631	261	1.06	0.91–1.24	1.04	0.88–1.22
Large rural centres	461	338	0.6	0.52–0.69	0.61	0.52–0.72
Other rural	1554	876	0.78	0.71–0.86	0.83	0.75–0.92
Remote	98	97	0.44	0.33–0.59	0.47	0.35–0.64
Missing	70	30	1.02	0.67–1.57	1.1	0.70–1.74
Year of the first trastuzumab dispensing						
2007–2008	1427	926	1		1	
2009–2010	1872	951	1.28	1.14–1.43	1.28	1.14–1.44
2011–2012	2147	921	1.51	1.35–1.69	1.55	1.38–1.74
2013–2014	2572	1087	1.54	1.38–1.71	1.62	1.45–1.81

### Chemotherapy

Among patients initiating adjuvant trastuzumab between 1 January 2007 and 30 June 2016, 14,079 (96.1%) were dispensed at least one dose of concomitant chemotherapy (Table 3). Of these, nearly all received a taxane-based regimen, with (48.4%) or without an anthracycline (47.3%). Patients dispensed anthracyclines and taxanes were younger than those receiving non-anthracycline taxane-based regimens (mean 52.6 vs. 57.9 years). Among patients with at least 540 days of follow-up, we did not observe an association between receiving anthracycline and likelihood of completing 12 months of trastuzumab (HR 0.95 for anthracycline- and taxane-based regimens vs. non-anthracycline taxane-based regimens, 95% CI 0.87–1.04).

**Table 3:** Chemotherapy regimens based on dispensings between 120 days before and 60 days after trastuzumab initiation (*n* = 14,644)

	Frequency	(%)
No chemotherapy drugs dispensed	565	4.9
At least one chemotherapy drug dispensed	14,079	96.1
Anthracycline- and taxane-based	6809	46.5
AC-TH	3287	22.4
FEC-DH	1561	13.7
Other	1961	13.4
Taxane-based (no anthracycline)	6664	45.5
TCH	3293	22.5
Paclitaxel–trastuzumab	1517	10.4
Docetaxel–trastuzumab	586	4
Other		
Anthracycline-based (no taxane)	426	2.9
No anthracycline or taxane	180	1.2
CMF-H	25	0.2
Other	155	1.1

*AC-TH* doxorubicin, cyclophosphamide, taxane and trastuzumab, *FEC-DH* 5-fluorouracil, epirubicin, cyclophosphamide, docetaxel and trastuzumab, *TCH* docetaxel, carboplatin and trastuzumab, *CMF-H* cyclophosphamide, methotrexate, 5-fluorouracil and trastuzumab

### Endocrine therapy

More than half of patients (*n* = 8,444, 57.7%) received at least one dispensing of endocrine therapy. The mean ages of endocrine therapy recipients and non-recipients were similar (54.8 vs. 55.9 years).

### Outcomes

#### Overall survival

**Fig. 1:**
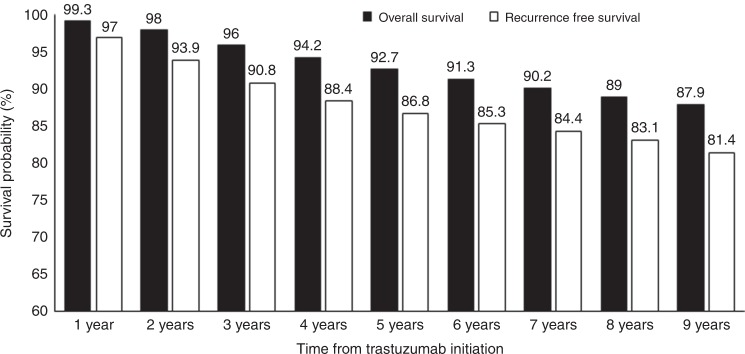
Annual overall and recurrence-free survival probabilities (*n* = 14644) of patients still in follow-up

By 31 May 2017, there were 1044 deaths (7.1%) among 14,644 patients initiating trastuzumab between 1 January 2007 and 30 June 2016, after median follow-up time of 4.7 years. The annual OS probabilities for this cohort are presented in Fig. 1.

In a multivariable model examining baseline characteristics, older age at the first trastuzumab dispensing, socioeconomic disadvantage and earlier year of trastuzumab initiation were associated with poorer survival (Table 4).

**Table 4:** Baseline characteristics associated with overall survival (*n* = 14644)

	Univariate analysis		Multivariable analysis	
	Hazard ratio	95% CI	Hazard ratio	95% CI
Age category				
≤30 years	1		1	
31–40 years	0.81	0.45–1.46	0.81	0.45–1.46
41–50 years	0.69	0.39–1.21	0.68	0.39–1.19
51–60 years	0.79	0.45–1.37	0.76	0.44–1.33
61–70 years	1.16	0.67–2.03	0.13	0.65–1.98
71–80 years	2.05	1.17–3.61	2.07	1.17–3.63
80+ years	6.11	3.35–11.14	6.45	3.53–11.78
Remoteness				
Capital cities	1			
Other metropolitan centres	1.04	0.82–1.32		
Large rural centres	1.11	0.87–1.41		
Other rural	1.15	0.99–1.33		
Remote	0.92	0.54–1.57		
Missing	1.32	0.73–2.40		
State				
New South Wales	1			
Australian Capital Territory	1.23	0.76–2.01		
Northern Territory	0.63	0.28–1.42		
Queensland	0.9	0.76–1.07		
South Australia	1.08	0.86–1.36		
Tasmania	0.96	0.60–1.55		
Victoria	0.97	0.83–1.14		
Western Australia	0.76	0.58–0.98		
Index of relative socioeconomic disadvantage				
1 (most disadvantage)	1		1	
2	0.97	0.08–1.19	0.95	0.78–1.15
3	0.78	0.63–0.97	0.76	0.62–0.94
4	0.8	0.66–0.98	0.78	0.64–0.96
5 (least disadvantage)	0.63	0.51–0.78	0.6	0.48–0.74
Missing	1.56	0.64–3.80	1.75	0.72–4.27
Year of the first dispensing				
2007–2008	1		1	
2009–2010	1.04	0.88–1.23	0.99	0.84–1.16
2011–2012	0.86	0.71–1.03	0.79	0.66–0.95
2013–2014	0.91	0.74–1.12	0.78	0.63–0.95
2015–2016	0.91	0.64–1.29	0.74	0.52–1.05

#### Survival by completion of trastuzumab course

In our multivariable analysis, excluding 133 patients who died within 540 days of their first dispensing of trastuzumab, not completing 12 months of adjuvant trastuzumab was associated with shorter OS (HR 1.41, 95% CI 1.23–1.62), after adjusting for age, socioeconomic disadvantage and year of trastuzumab initiation. Five- and 9-year survival probabilities were 94.6 and 90.2% among patients who completed 12 months of trastuzumab, and 91.8 and 86.2% among those who did not.

#### Recurrence

By 30 June 2016, 1,052 patients (7.2%) received at least one dispensing of trastuzumab for metastatic HER2-positive breast cancer. Figure 1 describes the annual RFS rates for patients commencing trastuzumab between 2007 and 2016.

**Fig. 2:**
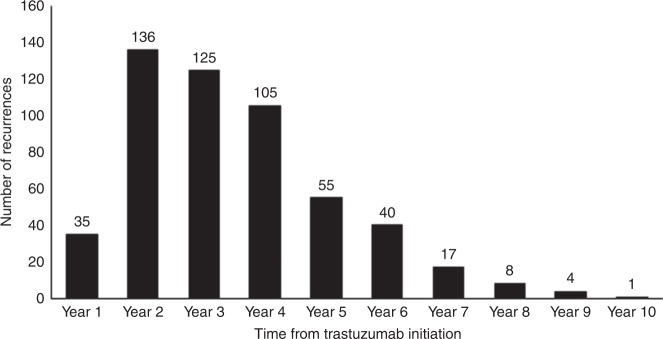
Number of recurrences by year of follow-up for patients with at least 5 years of follow-up (*n* = 5926)

Among patients with at least 5 years of follow-up (*n* = 5,926), 526 had a cancer recurrence (8.8%). Median duration of follow-up was 6.9 years (IQR 5.7–8.1 years). The number of recurrences in each year of follow-up is described in Fig. 2.

## Discussion

We report on nearly 15,000 patients who received trastuzumab for non-metastatic breast cancer in Australia between 2007 and 2016; to our knowledge, this is the largest cohort of HER2-positive breast cancer patients studied in a population-based setting. Survival outcomes in this observational study are similar to those reported in the landmark RCTs in HER2-positive early breast cancer.^4,6,10^ However, in contrast to some clinical trials, only two-thirds of patients initiating trastuzumab in our study completed the standard 12-month course.

This study illustrates the differences between RCT populations and patients treated in clinical practice and highlights the value of real-world studies in verifying and complementing clinical trial findings. The proportion of patients aged ≥60 years is twice as high in our study (33%) as in seminal RCTs of adjuvant trastuzumab (16%),^4,6^ confirming underrepresentation of older patients in clinical trials.^25,26^ The 12-month trastuzumab completion rate of 67.2% in our study was similar to the treatment completion rate of 68.6% in the NCCTG N9831/NSABP B-31 trials,^4^ but lower than 91.5% reported in the HERA trial.^6^ In our study, patients who did not complete 12 months of trastuzumab were more likely to be older, be socioeconomically disadvantaged and reside in rural and remote areas, all groups underrepresented in clinical trials.^27,39,40^

Despite lower completion rates, the outcomes of our study are comparable to landmark clinical trials; the 5-year OS rate in our study is 92.7%, compared with the 4-year OS rate of 89.3% in the HERA trial^8^ and 5-year OS rates of 91–92% in the BCIRG 006 trial.^10^ Our 9-year OS rate (87.9%) was similar to the 10-year OS (84%) reported in the NCCTG N98931/NSABP B-31 trials.^5^ OS estimates in our study are probably ameliorated by the absence of requirements for minimum tumour size or nodal status for accessing trastuzumab for HER2-positive early breast cancer in Australia, whereas most RCTs recruited only patients with tumours that were lymph node positive and/or larger than 1–2 cm.^4,6,10^ Therefore, we hypothesise that patients in our study were more likely to have cancers with favourable prognostic features than those in clinical trials, a finding that has been reported in previous real-world studies.^35,41,42^ The introduction of novel HER2-targeted agents for patients with recurrent metastatic disease probably also improved OS rates in our study, particularly for patients diagnosed in later years.

Patients who did not complete 1 year of trastuzumab had a 41% increased risk of death compared with those who did (HR 1.41), although the absolute difference between the groups is small (9-year OS rate 86.2 vs. 90.2%). The difference in survival associated with completing a year of trastuzumab in our study is larger than that reported in a meta-analysis of RCTs comparing 12 months with shorter durations of trastuzumab (HR 1.16).^43^ This is probably due to differences between clinical trial and real-world populations, as well as unmeasured confounders in our study, such as baseline cardiac impairment, that increase the likelihood of both early treatment cessation and death.

We report 4- and 5-year RFS rates of 88.4 and 86.8%, respectively. These figures may underestimate true recurrence rates as they are based on the receipt of trastuzumab for metastatic disease as a surrogate for recurrence. However, they are not dissimilar to 4-year DFS rates of 78.6–85.3% and 5-year DFS rates of 81–84% reported in clinical trials.^8,10,44^ Among patients with ≥5 years of follow-up, 87% of all recurrences occurred within 5 years of initiating adjuvant trastuzumab (Fig. 2). The majority of patients (70%) who started trastuzumab for metastatic disease did so during years 2–4 after their first dose of adjuvant trastuzumab. This suggests that the first 3 years after completing adjuvant trastuzumab are the most clinically relevant time period for monitoring and detecting recurrent metastatic disease in HER2-positive breast cancer.

When novel treatments are considered for public reimbursement, policy makers need reliable data on estimated treatment uptake and efficacy to support decision-making. Therefore, lower-than-expected rates of trastuzumab completion in routine practice have implications for the real-world applicability and assessment for reimbursement of novel HER2-targeted agents, such as neratinib, that are intended for use after patients complete 12 months of adjuvant trastuzumab.^45^ Patients in remote regions were 53% less likely to complete treatment compared with patients in capital cities, and those with the least socioeconomic disadvantage were 23% more likely to complete treatment than those with the most disadvantage. Therefore, geographical and financial barriers may limit patients’ ability to complete recommended treatment and potentially compromise cancer outcomes, although remoteness was not associated with survival in our study.

The major strength of our study lies in our ability to use population-wide medicines dispensing and survival data from a national, representative, cohort across all age groups, not just over 65s. However, the trade-off in utilising population-wide health administrative data is the lack of detailed clinical information, which contrasts with studies that have this information but are most commonly conducted in smaller and not necessarily generalisable populations. Our study is limited by the lack of clinical details such as tumour size and stage and patient comorbidities, including pre-existing cardiac disease. We did not have information on cause of death and therefore examined OS only. We also did not have access to clinical records documenting reasons for non-completion of 12 months of trastuzumab or exact dates or sites of relapse of metastatic disease.

## Conclusion

Although HER2-positive early breast cancer patients in the community are less likely to complete a 12-month course of adjuvant trastuzumab than clinical trial counterparts, survival and recurrence rates in this real-world population are reassuringly comparable to those reported in landmark clinical trials. Real-world studies of cancer medicine utilisation and outcomes provide valuable data to substantiate clinical trial results and support clinical and policy decision-making.

## Supplementary information

Supplementary Table 1

## Data Availability

We thank the Australian Government Department of Human Services for providing the data. Data access was granted by the Australian Government Department of Human Services External Request Evaluation Committee (Approval Numbers: MI1474, MI1475, MI1477 and MI5858). Access to the datasets analysed during this study is not permitted without the express permission of the approving human research ethics committees and data custodians.
