# Correction: Treatment patterns and survival in HER2-positive early breast cancer: a whole-of-population Australian cohort study (2007–2016)

**DOI:** 10.1038/s41416-020-0908-5

**Published:** 2020-05-29

**Authors:** Monica Tang, Andrea Schaffer, Belinda E. Kiely, Benjamin Daniels, Robert J. Simes, Chee K. Lee, Sallie-Anne Pearson

**Affiliations:** 1 Centre for Big Data Research in Health, University of New South Wales, Level 4, Lowy Building (C25), Corner Botany and High Streets, UNSW, Sydney, NSW 2052 Australia; 2 NHMRC Clinical Trials Centre, University of Sydney, Levels 4-6 Medical Foundation Building, 92-94 Parramatta Rd, Camperdown, NSW 2050 Australia

**Keywords:** Breast cancer, Targeted therapies, Translational research

Correction to: *British Journal of Cancer* (2019) **121**, 904–911; 10.1038/s41416-019-0612-5, published online 1 November 2019

Since the publication of this paper the authors noticed that an error was made in coding some trastuzumab doses as being dispensed for metastatic disease, when they were actually dispensed for early stage disease. This affected the calculation of the overall recurrence rate and yearly disease-free survival rates, but does not alter the conclusions of the paper. The adjusted numbers differ from the published results by 0.1–0.2%. This has been corrected below.

Recurrence

By 30 June 2016, 1027 patients (7.0%) received at least one dispensing of trastuzumab for metastatic HER2-positive breast cancer. Figure 1 describes annual RFS rates for patients commencing trastuzumab between 2007 and 2016.

**Fig. 1:**
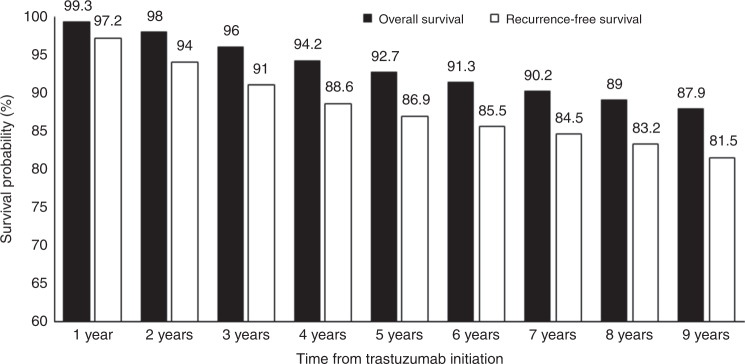
Annual overall and recurrence-free survival probabilities (*n* = 14644) of patients still in follow-up.

